# Microfluidic Electrospray Niacin Metal-Organic Frameworks Encapsulated Microcapsules for Wound Healing

**DOI:** 10.34133/2019/6175398

**Published:** 2019-04-22

**Authors:** Guopu Chen, Yunru Yu, Xiuwen Wu, Gefei Wang, Guosheng Gu, Feng Wang, Jianan Ren, Huidan Zhang, Yuanjin Zhao

**Affiliations:** ^1^Research Institute of General Surgery, Jinling Hospital, Medical School of Nanjing University, Nanjing 210002, China; ^2^State Key Laboratory of Bioelectronics, School of Biological Science and Medical Engineering, Southeast University, Nanjing 210096, China; ^3^School of Engineering and Applied Sciences and Department of Physics, Harvard University, Cambridge, Massachusetts 02138, USA

## Abstract

Niacin metal-organic frameworks (MOFs) encapsulated microcapsules with alginate shells and copper-/zinc-niacin framework cores were* in situ* synthesized by using a microfluidic electrospray approach for wound healing. As the alginate shells were bacteria-responsively degradable, the niacin MOFs encapsulated microcapsules could intelligently, controllably, and programmably release calcium, copper, and zinc ions, depending on the degree of infections. The released ions could not only kill microbes by destroying their membrane and inducing the outflow of nutrient substance, but also activate copper/zinc superoxide dismutase (Cu/Zn-SOD) to eliminate oxygen free radicals and rescue the cells from oxidative stress injury. Furthermore, the simultaneously released niacin could promote hemangiectasis and absorption of functional metal ions. Thus, the niacin MOFs encapsulated microcapsules were imparted with outstanding antibacterial, antioxidant, and angiogenesis properties. Based on an in vivo study, we have also demonstrated that the chronic wound healing process of an infected full-thickness skin defect model could be significantly enhanced by using the niacin MOFs encapsulated microcapsules as therapeutic agent. Therefore, the microfluidic electrospray niacin MOFs encapsulated microcapsules are potential for clinical applications.

## 1. Introduction

Chronic hard-to-healing wounds treatment and their therapeutic impediments continue to be fundamental healthcare concern and contribute to increasing economically healthcare burden worldwide [1–4]. The regulatory costs and development time to market can be significantly reduced when a medical therapeutic product is identified as a medical device [5–10]. Hence, nonbiological and nonpharmacological strategies are more appealing in this regard, and several approaches have been developed, including suction devices, metal ions, and small organic compounds [11–14]. Of particular concern is the use of copper ion (Cu^2+^) and zinc iron (Zn^2+^) as they are essential elements widely used in humans and have been identified to promote wound healing through angiogenesis stimulation, collagen deposition, and antioxidation [15, 16]. Although multiple applications of copper/zinc salts and oxide could improve the therapeutic effects, patients are in the face of the increasing risk of copper/zinc ions toxicity, which can interfere with the homeostasis of calcium ion, damage DNA, and generate reactive oxygen species [17–19]. Thus, the controlled slow release of copper/zinc ions is required to reduce the risk of ion toxicity.

Metal-organic frameworks (MOFs) are a class of coordination crystalline polymers consisting of metal ions and polydentate organic ligands that interact to form clusters [20, 21]. They have been extensively investigated for applications in chemistry, environment, energy, sensing, and biomedicine [22–24]. Although MOFs can be employed to store and release copper/zinc ions based on their tunable chemical and physical properties, the instability of the copper based-MOFs (Cu-MOFs) or zinc based-MOFs (Zn-MOFs) in protein-contained solutions, such as tissue fluids, could restrain their usefulness in wound healing [25–27]. Thus, stratagems of coating or encapsulating of hydrogels have been developed to improve their stability and address the problem [28–30]. However, when controllable and programmable releases are needed, the random encapsulation and distribution of MOFs in the hydrogels make them difficult for practical applications. In addition, the debatable biocompatibility of the organic ligands has greatly affected their biomedical values. Therefore, the development of biocompatible MOFs encapsulated hydrogel with designed controllable release is still anticipated.

**Figure 1:**
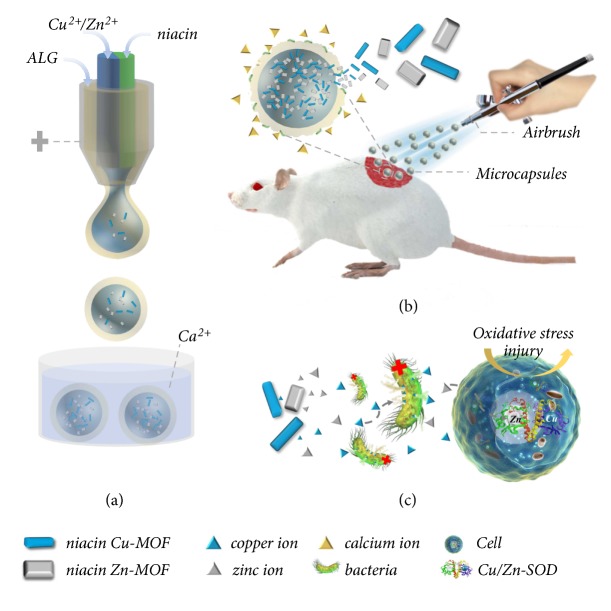
Schematic illustrations of the preparation and application of the niacin MOFs encapsulated microcapsules. (a) The microcapsules generation process from the microfluidic electrospray. (b) The niacin MOFs encapsulated microcapsules applied to wound healing by airbrush. The alginate shell could be degraded by bacteria and body fluid, which released calcium ions and accelerated the release of MOFs. (c) The antibacterial and antioxidant capabilities of the released copper and zinc ions.

In this paper, we present a novel niacin MOFs encapsulated hydrogel microcapsule with the desired feature by using a microfluidic electrospray, as schemed in Figure 1. Benefiting from the facile device, high production, and ability to precisely control the monodispersity, size, structure, and component of their generated emulsion templates, microfluidic electrospray has emerged as a promising and versatile technique for fabricating functional microparticles or microcapsules for different applications [31–37]. However, these approaches have not been applied to synthesize and encapsulate multiple MOFs, and the potential value of the MOFs encapsulated microcapsules for biomedical engineering has not been investigated. Thus, we herein fabricated the desired microcapsules by using a double-emulsion capillary microfluidic electrospray system (Figure 1(a)). The alginate hydrogel shells of the microcapsules were formed by immediate gelation reaction between sodium alginate (ALG) and calcium ions, while the MOFs cores were* in situ* synthesized in the alginate shells when the copper/zinc ions and niacin solutions were injected and merged from the microfluidic channels. As the alginate shells were bacteria-responsive degradable, the resultant microcapsules could realize intelligent, programmable, and sustainable release of multifunctional calcium, copper, and zinc ions, which were with antibacterial, antioxidant, and angiogenesis properties (Figures 1(b) and 1(c)). What is more, the organic ligand niacin released synchronously has been widely used in clinic medicine and food additives, which could promote blood circulation and the uptake of metal ions. Thus, the niacin MOFs encapsulated microcapsules showed distinctive functions in chronic wound healing.

## 2. Results and Discussion

### 2.1. Synthesis of the Niacin MOFs Encapsulated Microcapsules

**Figure 2:**
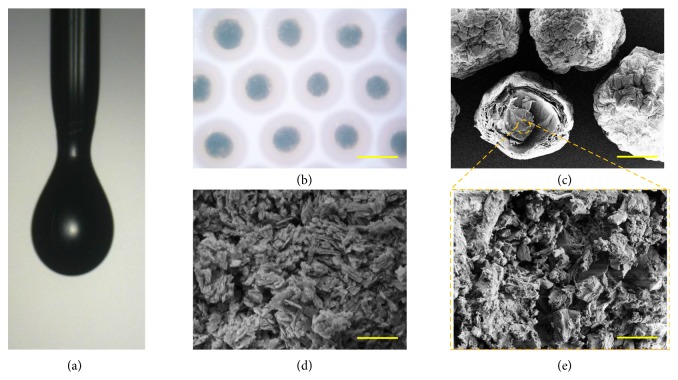
The preparation of the niacin Cu-MOFs encapsulated microcapsules. (a) The real-time image of the microfluidic electrospray process of the microcapsules. (b) Microscope image of the microcapsules. (c–e) Scanning electron microscope (SEM) images of the (c) niacin Cu-MOFs encapsulated microcapsules, (d) niacin Cu-MOFs, and (e) niacin Cu-MOFs inside the microcapsules. Scale bar in (b) is 300*μ*m, in (c) is 100*μ*m, and in (d, e) is 5*μ*m.

In a typical experiment, the biomedical hydrogel microcapsules with core-shell structures were made from a double-emulsion capillary microfluidic electrospray system. The double-emulsion microfluidic system was assembled by aligning an inner theta capillary into a tapered capillary. The inner phase of the niacin solution and the zinc acetate (Zn(CH_3_COO)_2_) solution or copper acetate (Cu(CH_3_COO)_2_) solution precursor flowed inside each barrel of the inner theta capillary, and the outer phase of ALG precursor was pumped along the outer tapered capillary. On account of the low Reynolds number and hydrodynamic focusing effect, the two inner MOFs precursors could form laminar flows and were sheathed by the outer alginate stream at the merging point of these fluids (Figure 2(a) and [Supplementary-material supplementary-material-1], Supplementary Materials). Then, the coflow was broken up into the droplets by the outer electric field and sprayed into the gelling pool which contained calcium chloride (CaCl_2_) solution. The fast diffusion of Ca^2+^ could instantly solidify the alginate to form the shell structure of the microcapsules. During this period, the inner Cu(CH_3_COO)_2_ or Zn(CH_3_COO)_2_ reacted with the excess niacin immediately to* in situ* form MOFs within alginate shells. Finally, the niacin MOFs encapsulated microcapsules could be collected in the CaCl_2_ solution for further strengthening the alginate gelation (Figures 2(b)–2(e) and [Supplementary-material supplementary-material-1], Supplementary Materials). By dynamically regulating the strength of the electric field, the size of the microcapsules could be precisely controlled ([Supplementary-material supplementary-material-1], Supplementary Materials).

The successful preparation of the MOFs encapsulated in the microcapsule core was evaluated by Fourier Transform Infrared Spectroscopy (FTIR) analysis and X-ray diffraction (XRD) ([Supplementary-material supplementary-material-1]). The characteristic peaks of copper- and zinc-MOFs could be observed (1359, 1626 cm^−1^(COO^−^), 1651 cm^−1^ (skeleton of pyridine ring), 3043, 3091 cm^−1^(C=N)), which confirmed the successful reaction between copper or zinc ions and niacin ligands (Figures [Supplementary-material supplementary-material-1], [Supplementary-material supplementary-material-1]). The crystal structure of the MOFs was further studied by X-ray powder diffraction (XRD). The copper- or zinc-MOFs wrapped with calcium alginate exerted clear narrow and sharp peaks, which were similar to the copper- or zinc-MOFs alone, indicating a successful synthesis (Figures [Supplementary-material supplementary-material-1], [Supplementary-material supplementary-material-1]). The resultant niacin MOFs encapsulated microcapsules were imparted with the function of highly controlled release because of the protective and sustainable alginate shell. The metal ions released from the MOFs encapsulated microcapsules were observed to have a significantly mitigated process compared to that of the niacin MOFs contacting with the phosphate-buffered saline (PBS) solution ([Supplementary-material supplementary-material-1]). The porosity of the synthesized MOFs was not studied in this study as their functions on wound healing process were not related to their porosity.

### 2.2. Biomedical Properties of the Niacin MOFs Encapsulated Microcapsules

To investigate the biomedical application potential of the niacin MOFs encapsulated microcapsules, their biocompatibility was estimated firstly. Microcapsules with different amounts of niacin MOFs could be fabricated by changing the concentration of the metal ion precursor. Then, 3T3 mouse fibroblasts were used to assess the cytotoxicity of the microcapsules through the extract solution method and cell counting kit-8 (CCK-8) assay ([Supplementary-material supplementary-material-1]). The microcapsules showed increased cytotoxicity with increased metal ion concentrations, while the fibroblasts proliferated slightly when the metal ions concentration was very low. This should be ascribed to the beneficial stimulation effects of the metal ions together with the great biocompatibility of the niacin, and certain dose of copper/zinc ions can promote cell proliferation. Thus, based on the features of sustainable and controllable release of the MOFs, the microcapsules could be applied to cell cultures with improved viability. The concentration of metal ions was set at 0.1 mM for the follow-up experiments to ensure the high biocompatibility for the potential clinical application.

**Figure 3:**
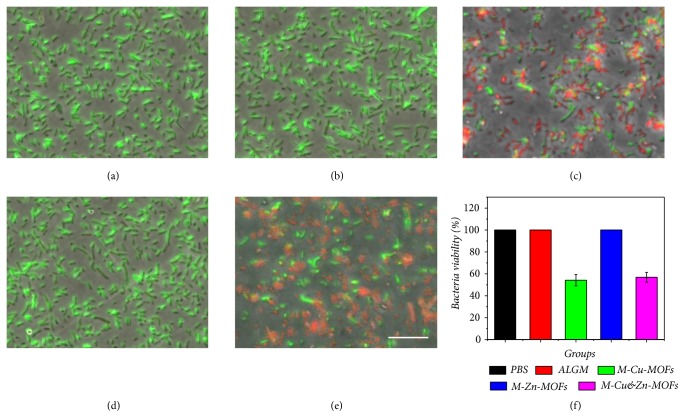
The antibacterial activities of the microcapsules with different niacin MOFs encapsulated. (a–e) The confocal laser scanning images of the Live/Dead fluorescent stained bacteria on (a) PBS, (b) ALG hydrogel, and (c–e) M-Cu-MOFs, M-Zn-MOFs, and M-Cu&Zn-MOFs. (f) The statistical Gram of the bacteria viability of microcapsules with different niacin MOFs encapsulated. Scale bar in (e) is 10*μ*m.

In practical situations, wounds are easily infected by the gathering of bacteria in a dirty environment, which may trigger an inflammation or immune response, impede the healing process, and even cause life-threatening complications. Hence, the antibacterial capability of the niacin MOFs encapsulated microcapsules was also investigated. Gram-negative* Escherichia coli*. (*E. coli*) that accounts for most clinical wound infections was employed to perform the antibacterial test. The testing samples were divided into five groups, namely, phosphate buffer saline (PBS), simple alginate microcapsules (ALGM), niacin Cu-MOFs encapsulated microcapsules (M-Cu-MOFs), niacin Zn-MOFs encapsulated microcapsules (M-Zn-MOFs), and both the niacin Cu-MOFs and Zn-MOFs encapsulated microcapsules (M-Cu&Zn-MOFs). It was found from the Live/Dead bacterial staining that the bacteria were alive and maintained their original clavate appearance in the groups of PBS, ALGM, and M-Zn-MOFs (Figures 3(a), 3(b), 3(d), and 3(f)). However, with the appearance of Cu-MOFs, the niacin MOFs encapsulated microcapsules showed satisfactory antibacterial effect after contacting with the bacteria in groups M-Cu-MOFs and M-Cu&Zn-MOFs (Figures 3(c), 3(e), and 3(f)). The antibacterial property of the MOFs encapsulated microcapsules could be related to copper ions' inherent ability to destroy the membrane of the microbes, causing outflow of inside nutrient substance, which leads to the death of microbes finally.

**Figure 4:**
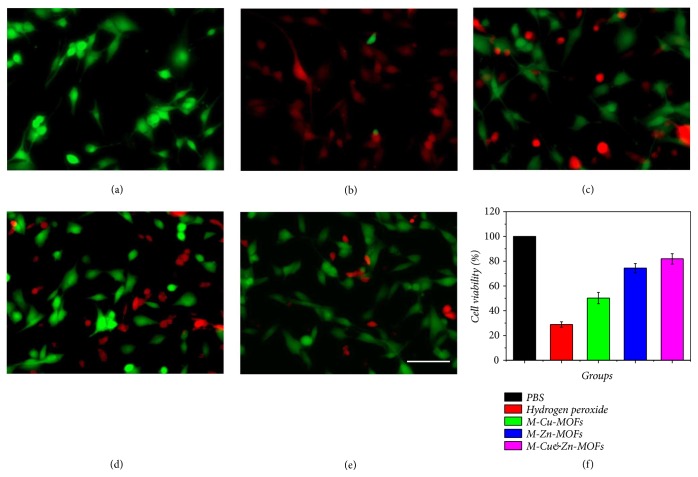
The antioxidant activity tests of the microcapsules with different niacin MOFs encapsulated. (a–e) The confocal laser scanning images of the Live/Dead fluorescent stained fibroblasts treated with (a) PBS, (b) 0.04% hydrogen peroxide, and (c–e) 0.04% hydrogen peroxide coupled with M-Cu-MOFs, M-Zn-MOFs, and M-Cu&Zn-MOFs. (f) The statistical Gram of the cell viability of microcapsules with different niacin MOFs encapsulated after treating with hydrogen peroxide. Scale bar in (e) is 10*μ*m.

Oxidative stress injury after wound seriously aggravates edema, shock, and inflammation in the wound bed, inhibits the migration and proliferation of tissue repair cells, and as a result influences wound healing process. Therefore, the antioxidant capability of the niacin MOFs encapsulated microcapsules was also measured in this study, where hydrogen peroxide was employed to stimulate the oxidative stress injury. The testing groups were divided into five groups, namely, PBS, hydrogen peroxide, and hydrogen peroxide coupled with M-Cu-MOFs, M-Zn-MOFs, and M-Cu&Zn-MOFs. The 3T3 fibroblasts died massively after contacting with hydrogen peroxide compared with the PBS group (Figures 4(a), 4(b), and 4(f)), indicating a serious oxidative stress injury. However, with the existence of niacin MOFs encapsulated microcapsules, the fibroblasts could still keep high cell viability after contacting with hydrogen peroxide, especially with the existence of niacin Zn-MOFs. This phenomenon should be attributed to the fact that the copper and zinc ions can activate the copper/zinc superoxide dismutase (Cu/Zn-SOD) in cytoplasm of the cells, which is regarded as the top natural enemy of oxygen free radicals as it can eliminate oxygen free radicals, block the damage of oxygen free radicals to cells, and repair the damaged cells in time.

Though the antibacterial and antioxidant capabilities of the niacin MOFs encapsulated microcapsules had been demonstrated, different wounds faced different degree of infections and oxidative free radical injury. Thus, the responsive release of metal ions to different number of bacteria and degree of infection in the wound bed was needed in practical applications. It is worth mentioning that the sodium alginate was recognized as dietary fiber with the ability to be degraded by bacteria. Hence, the bacteria-responsive degradation capability of microcapsules was evaluated. The degradation rate of the microcapsules increased with increased concentration of the bacteria ([Supplementary-material supplementary-material-1]). It could be demonstrated that when large number of bacteria gathered at the wound bed, the degradation rate of the alginate shell of the microcapsules accelerated. During this process, a mass of calcium ions were released into the wound followed by large amount of niacin MOFs outflowing from the core, which accelerated the release of copper and zinc ions to play an enhanced antibacterial and antioxidant role. As a result, the niacin MOFs encapsulated microcapsules could realize intelligent, controllable, and programmable release of calcium, copper, and zinc ions to promote wound healing.

### 2.3. The Niacin MOFs Encapsulated Microcapsules for Wound Healing

**Figure 5:**
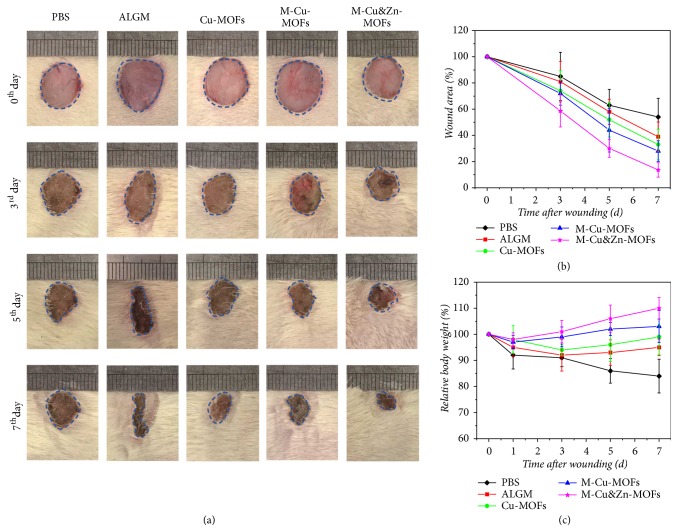
Evaluation of the MOFs encapsulated microcapsules on wound healing in infected full-thickness skin defect model. (a) Representative digital photos of the skin wounds treated with PBS, ALGM, Cu-MOFs, M-Cu-MOFs, and M-Cu&Zn-MOFs. (b) The statistical Gram of the mean wound area. (c) Quantitative analysis of the relative body weight.

**Figure 6:**
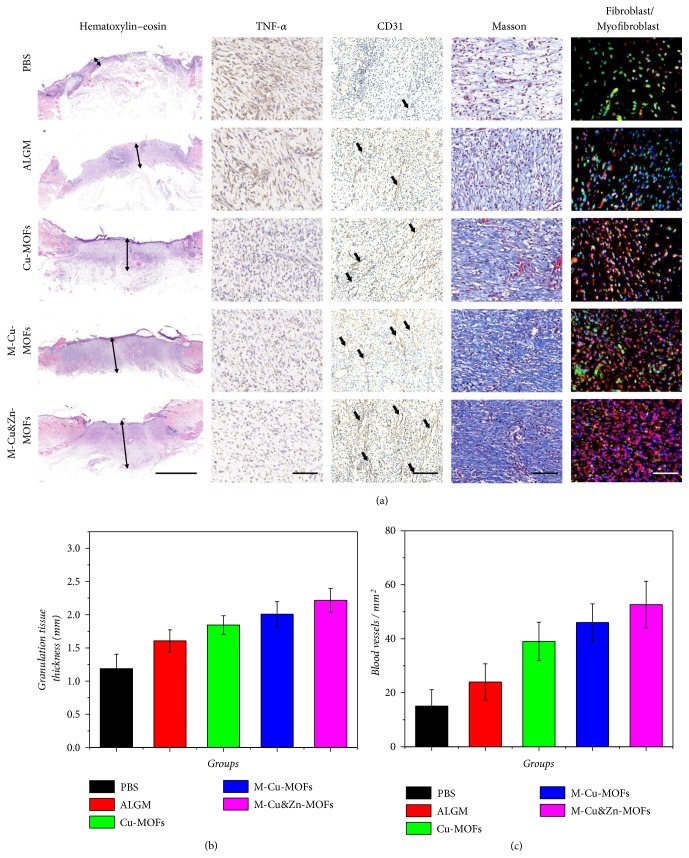
Biological mechanism of the wound healing process. (a) Hematoxylin-eosin staining, IHC staining of TNF-*α* and CD31, Masson trichrome stain, and double immunofluorescent staining of fibroblast marker vimentin (green) and myofibroblast marker *α*-SMA (red) of granulation tissues in the wound bed after 7 d at low magnification. (b) Quantitative analysis of granulation tissue thickness. (c) Quantification of CD31 labeled structures. Scale bar in (a) is 2mm in hematoxylin-eosin staining, 100*μ*m in IHC staining of TNF-*α*, Masson trichrome stain, and double immunofluorescent staining, and 200*μ*m in IHC staining of CD31.

The* in vivo* experiment was finally conducted to investigate the practical value of niacin MOFs encapsulated microcapsules in chronic hard-to-healing wound healing on the basis of their desirable biocompatibility and antibacterial and antioxidant capabilities. An* E. coli* infected full-thickness skin defect model on the back of a mouse with a diameter of about 1 cm was constructed. The testing samples were divided into five groups, namely, the PBS, ALGM, Cu-MOFs, M-Cu-MOFs, and M-Cu&Zn-MOFs. The wound closure processes and healing abilities in these groups were all recorded for detailed analyses. Wounds treated with MOFs encapsulated microcapsules were almost healed after 7 days, whereas healing in the wound treated with ALGM, Cu-MOFs needed more time (Figure 5(a)). The quantitative analyses of wound closure rates, body weight over time, and granulation tissue thickness also showed that wounds treated with M-Cu&Zn-MOFs experienced a significantly shorter healing period and better physical condition than other groups (Figures 5(b), 5(c), 6(a), and 6(b)). The capability of M-Cu&Zn-MOFs to promote wound healing* in vivo *may be attributed to the moist environment provided by the microcapsules, and the intelligent, programmable, and sustained release of noncytotoxic amounts of calcium, copper, and zinc ions. The calcium ions released from the alginate shells could promote angiogenesis and play important role in the skin barrier regeneration in the early wound healing process. Then, as the alginate shells degraded by enzymes and bacteria in the wound bed, the copper and zinc ions from the cores were released quickly and played many unique roles. Copper ions could induce anti-infection, angiogenesis, and collagen deposition, whereas zinc ions are components of over several metal binding enzymes which participate in the regeneration of matrix in the wound bed, and the copper or zinc ions could both activate the Cu/Zn-SOD to reduce oxidative stress injury. The core-shell structure of the microcapsules could reduce the mutual interference of the multiple metal ions to a certain degree and help to balance the concentration of the ions in the wound bed.

As infection is one of the leading causes of death for traumatic patients, the immunohistochemistry (IHC) analysis of the secretion of the typical proinflammatory factor tumor necrosis factor-*α* (TNF-*α*) in the wound bed was employed to evaluate the efficacy of the niacin MOFs encapsulated microcapsules in preventing infection (Figure 6(a)). After one week of healing, large amount of TNF-*α* was detected in the control group of simple alginate hydrogel, which suggested a severe inflammatory response. On the contrary, a small amount of secretion was observed in the niacin MOFs encapsulated groups, especially the M-Cu&Zn-MOFs group, indicating that there were few signs of inflammation. This phenomenon should be contributed to the gentle and sustainable release of the metal ions in microcapsules, which demonstrated their distinct antibacterial and antioxidant functions for wound healing.

To further investigate the biological mechanism of the healing process, the degree of angiogenesis and collagen deposition was evaluated. Activation of angiogenesis is required to sustain newly regenerated granulation tissue; therefore, the correction of impaired local angiogenesis is very important for the treatment of chronic wounds. From the microscale observation based on the CD31 staining, it was found that the blood vessel density in the wound bed was clearly increased in the Cu-MOFs, M-Cu-MOFs, and M-Cu&Zn-MOFs treated mice (Figures 6(a) and 6(c)). The capacity of these groups to stimulate new blood vessel formation may be attributed to copper ions, which has been identified to increase the expression of angiogenic genes for PDGF, bFGF, and VEGF. As a result, the acceleration of angiogenesis was likely to allow an adequate supply of nutrients and oxygen, as well as expediting migration of the humoral factors and requisite cells into the wounds and then improving the generation of collagen and granulation tissue, leading to facilitated wound healing. The synthesis and deposition of collagen also play an important role in tissue repair remodeling during the wound healing process. As a cofactor to lysyl oxidase, copper ions could stimulate the secretion of matrix metalloproteinase-2 (MMP-2) and collagen in fibroblasts. On the other hand, the zinc ions could protect the fibroblasts from oxidative stress injury and reduce the degradation of collagen. Masson's trichrome staining was employed to evaluate collagen deposition and fibroblasts proliferation in the wound. Significantly more collagen deposition was observed in the wounds treated with Cu-MOFs, M-Cu-MOFs, and M-Cu&Zn-MOFs than in wounds treated with PBS and ALGM (Figure 6(a)). The facilitated synthesis of collagen is related to the promoted phenotypic differentiation of fibroblasts into myofibroblasts to a great extent. The myofibroblasts are very important in the matrix formation and remodeling phase during wound healing process through promoting collagen deposition and wound contraction. Thus, double immunofluorescent staining was performed to identify the ratio between fibroblasts (vimentin) and myofibroblasts (*α*-smooth muscle actin, *α*-SMA). The results showed that the number and ratio of myofibroblasts increased significantly in the Cu-MOFs, M-Cu-MOFs, and M-Cu&Zn-MOFs groups (Figure 6(a)), indicating a promotion of wound healing. These results demonstrated that the niacin MOFs encapsulated microcapsules were ideal materials for remodeling the granulation tissue and promoting wound healing through intelligent, programmable, and sustainable release of multifunctional calcium, copper, and zinc ions.

## 3. Conclusion

In summary, we have developed niacin MOFs encapsulated multifunctional microcapsules for chronic wound healing by using a microfluidic electrospray. A double-emulsion capillary microfluidic device was employed to fabricate the microcapsules with alginate shells and* in situ* synthesize copper- or/and zinc-MOFs cores. Because of the core-shell structure and bacteria-responsive degradation, the resultant niacin MOFs encapsulated microcapsules were endowed with the capability to intelligent, controllable and programmable release of calcium, copper and zinc ions. With a biomedical dose, the released copper ions could show satisfactory antibacterial property and the Cu/Zn-SOD could be activated with the release of zinc ions, exhibiting superior antioxidant capability. The practical value of the resulting niacin MOFs encapsulated microcapsules has been demonstrated through the in vivo study that the chronic wound healing process of an infected full-thickness skin defect model could be enhanced significantly though reducing inflammation, anti-bacteria, antioxidation, and promoting angiogenesis, collagen deposition and fibroblasts phenotypic differentiation. These features manifest that the niacin MOFs encapsulated microcapsules are efficient for chronic wound healing, and thus we believe that these niacin MOFs encapsulated microcapsules will be widely used in clinic based on their excellent capabilities.

## 4. Materials and Methods

### 4.1. Material, Cell Lines, and Animals

Copper acetate monohydrate and calcium chloride were bought from Alfa Aesar. Zinc acetate, nicotinic, and sodium alginate were purchased from Aladdin. The antibodies CD31, TNF-*α*, *α*-smooth muscle actin (*α*-SMA), vimentin, and NIH 3T3 cell lines were obtained from Nanjing Microworld Biotechnology Co., Ltd (China). The cells were cultured in Eagle's Minimum Essential Medium (Gibco, USA) supplemented with 10% fetal bovine serum (Gibco, USA) under the condition of 37°C and 5% CO_2_. The 8-12-week male Sprague-Dawley mice were obtained from Jinling Hospital. All animals were treated in strict accordance with the recommendations in the Guide for the Care and Use of Laboratory Animals of the National Institutes of Health, USA. All animals' experimental protocols and care were reviewed and approved by Animal Investigation Ethics Committee of the Jinling Hospital.

### 4.2. Double-Emulsion Microfluidic Device Design

The double-emulsion microfluidic device consisted of a glass slide, a tapered capillary (World Precision Instruments Inc.), a spindle theta capillary, and two spindle tapered capillaries. The outer glass capillary was tapered by capillary puller (P-97, Sutter Instrument) and sanded to reach an orifice diameter of 150*μ*m. The theta capillary and two cylindrical capillaries were pulled by a laboratory portable Bunsen burner (Honest Micro Torch) to form spindle tips. The spindle theta capillary was then coaxially inserted into the outer tapered capillary with two spindle capillaries inserted into each barrel as two inlets. A needle with two crevices at the bottom was used at the connection of the inner theta capillary and outer channel. Afterwards, a transparent epoxy resin (Devcon 5 Minute Epoxy) was used to seal where it is necessary.

### 4.3. Fabrication of the Niacin MOFs Encapsulated Microcapsules

The niacin MOFs encapsulated microcapsules were fabricated by using a double-emulsion microfluidic device, which was then integrated with the electrospray collection device. Voltages from a voltage generator were used to provide electric field. Due to the low Reynolds number and hydrodynamic focusing effect, the two precursors formed laminar flows and were sheathed by outer 2wt% sodium alginate stream. Afterwards, the coflow was broken up into microdroplets with the assistance of the outer electric field. The formed microdroplets were collected by using a container with 2wt% calcium chloride to allow the alginate solution to gel. The inner phases were 0.1 mmol/mL niacin and 0.01, 0.1, 1, 10, and 100 mM copper acetate or zinc acetate solutions, and 2wt% hydroxypropyl methylcellulose was added to increase the viscosity of the solutions. Since the concentration of niacin is much higher than the metal ion precursor solutions, the output of the MOFs depends on the concentration of different metal ion precursors. Because of the fast reaction between the two MOFs precursors, the MOFs could* in situ* form in the core. Thus, the niacin MOFs encapsulated microcapsules could be fabricated by this method. The size of microcapsules under different voltage, collecting distance, flow rate, and concentration was evaluated.

### 4.4. Characterization

Bright-field images of the niacin MOFs encapsulated microcapsules were recorded by microscopy (OLYMPUS IX71) equipped with CCD cameras (DP30BW). The microstructures of the niacin copper- and zinc-MOFs encapsulated microcapsules were characterized by a scanning electron microscope (SEM, HITACHI, S-3000N). The infrared spectra were performed by the Thermo Scientific Nicolet iS50 FTIR spectrometer. The X-ray diffraction (XRD) was performed to analyze the crystal structure of the niacin MOFs in the microcapsules core.

### 4.5. Niacin Release Test of the Microcapsules

The sodium alginate stream solution was pumped at the speed of 1mL/h, the niacin solution was pumped at the speed of 0.2mL/h, and different concentrations of the metal ion precursor solution were pumped at the speed pf 0.15 mL/h. All the fluids were flowed in the microfluidic device in 20 min. The resultant niacin MOFs encapsulated microcapsules were collected and washed by ethyl alcohol, water, and phosphate buffer saline (PBS) solution three times, respectively. Then, the collected microcapsules were incubated into 1mL PBS (pH7.4) and shaken with a speed of 300rpm at 37°C. At predetermined intervals, 100 *μ*L of released media was taken out for content measurement and replenished with an equal volume of fresh media at 37°C. The amount of released niacin was measured by UV-vis spectroscopy (Cary 60, Agilent Technologies).

### 4.6. Cytotoxicity Tests of the Niacin MOFs Encapsulated Microcapsules

The resultant niacin MOFs encapsulated microcapsules with different concentrations of metal ion precursor solution were added to each well containing 1mL culture medium and incubated for 24h. The 3T3 fibroblasts were plated in 96-well cell culture dishes with 4000 cells per well (100 *μ*L) for 24 h to allow attachment before the test. Afterwards, the media in the 96-well dish were removed, and the cells were rinsed by PBS. Different 24 h aged extract solutions were added to the wells and the cells were incubated with the extract solutions for another 24 h. The cell viability of the experimental groups was measured by a cell counting kit-8 test and expressed by the percentage of living cells with respect to the control group.

### 4.7. Antibacterial Capability of the Niacin MOFs Encapsulated Microcapsules

The Gram-negative* E. coli *was employed to investigate the antibacterial activities of the niacin MOFs encapsulated microcapsules. The control groups were PBS, simple alginate microcapsules (ALGM), niacin Cu-MOF-laden microcapsules (M-Cu-MOF), niacin Zn-MOF-laden microcapsules (M-Zn-MOF), and both the niacin Cu-MOF- and Zn-MOF-laden microcapsules (M-Cu&Zn-MOF). Firstly, tested groups of microcapsules were prepared according to the cytotoxicity tests to ensure the biocompatibility and then incubated in the 96-well plate with PBS (pH 7.4) for 1h at 37°C. Subsequently, 100 *μ*L of bacteria solution (1 x 10^4^ CFU mL^−1^) was added to the well and incubated with the microcapsules for 24h at 37°C after which the suspension was taken out. The suspension was then fluorescently imaged by a Live-Dead assay with two staining agents, NucView Green Live and propidium iodide. After rinsing with PBS three times, the stained bacteria were observed by Opera Phenix (PerkinElmer Inc., UK).

### 4.8. Antioxidant Capability of the Niacin MOFs Encapsulated Microcapsules

The hydrogen peroxide and 3T3 cells were employed to build the cellular oxidative stress injury models. The control groups were normal 3T3 cells and 3T3 cells treated with 0.04% hydrogen peroxide; the experiment groups were 3T3 cells treated with a mixed medium of 0.04% hydrogen peroxide and M-Cu-MOF, M-Zn-MOF, and M-Cu&Zn-MOF. The 3T3 cells were plated in 96-well cell culture dishes with 4000 cells per well (100 *μ*L) for 24 h to allow attachment before the test. Afterwards, the media in the 96-well dish were removed, and the cells were rinsed by PBS. The microcapsules were collected and washed by ethyl alcohol, water, and PBS solution three times, successively, and then they together with hydrogen peroxide were induced into the cells and cocultured for 4 h. The cell viability was measured by counting Kit-8 test, and the control group of normal 3T3 cells was set as 100%.

### 4.9. Bacteria-Responsive Degradation Test of the Microcapsule

The bacteria-responsive degradation test of the microcapsules was examined gravimetrically under simulated infected conditions. Briefly, the microcapsules were collected and washed by ethyl alcohol, water, and PBS solution three times, successively, and weighed (W_0_). Weight loss of W_0_ was monitored as a function of incubation time in different concentration of* E. coli* solution at 37°C. At predetermined intervals, the samples were carefully withdrawn from the bacteria solution. Then, the samples were freeze-dried and weighed (W_t_). The weight loss percentage (ΔW %) was defined as in the following equation.

(1) $$\begin{array}{c} \Delta W\left(\;\%\;\right)=\frac{\left(W_{0}-W_{t}\right)}{W_{0}}\times 100\% \end{array}$$

### 4.10. Chronic Wound Healing Study of the Niacin MOFs Encapsulated Microcapsules

A mouse* E.coli* infected full-thickness cutaneous wound model was employed to evaluate the effect of niacin MOFs encapsulated microcapsules on chronic wound healing. First, a total of 30 healthy Sprague-Dawley rats were anesthetized and their backs were shaved. A round full-thickness cutaneous wound (1 cm) area was created on the back of each mouse after which 200 *µ*L* E. coli* solution (1 × 10^8^ CFU/mL) was introduced onto the wound bed and then divided into five groups randomly. The five groups were treated with PBS, ALGM, Cu-MOFs, M-Cu-MOFs, and M-Cu&Zn-MOFs, respectively. The mixed solution of Milli-Q water and microcapsules was sprayed onto the wound surface by airbrush. Thereafter, the rats were housed in cages and allowed to heal for 7d, respectively. Photos of the wounds were taken at days 0, 3, 5, and 7. All the mice were sacrificed after 7d and granulation tissues over the wounds were excised. The piece was immersed in neutral formaldehyde for further immunohistochemistry and histology analysis.

#### 4.10.1. Histology and Immunohistochemistry

The granulation tissue samples were taken out from the neutral formaldehyde and then dehydrated and embedded in paraffin. Serial sections with the thickness of 5 *µ*m were acquired by a microtome and were prepared for hematoxylin-eosin, Masson trichrome, and immunohistochemical staining. Sections for immunohistochemistry were stained with TNF-*α*. For neovascularization evaluation, sections were stained with CD31. Sections for evaluation of fibroblast phenotypic differentiation were stained with vimentin and *α*-smooth muscle actin.

## Data Availability

All data needed to evaluate the conclusions in the paper are present in the paper. Additional data related to this paper may be requested from the authors.

## Supplementary Materials

Supplementary 1 Figure S1. The real-time images of the microfluidic electrospray process of the niacin MOFs encapsulated microcapsules.

Supplementary 2 Figure S2. The preparation of the niacin Zn-MOFs encapsulated microcapsules. (a) Microscope image of the microcapsules. (b-d) Scanning electron microscope (SEM) images of the (b) niacin Zn-MOFs encapsulated microcapsules, (c) niacin Zn-MOFs, and (d) niacin Zn-MOFs inside the microcapsules (indicated with yellow arrows). Scale bar in (a) is 300*μ*m, in (b) is 100 *μ*m, in (c) is 2 *μ*m, and in (d) is 10*μ*m.

Supplementary 3 Figure S3. The relationship between the microcapsule size and (a) voltage, (b) collecting distance, (c) flow rate, and (d) concentration of the alginate solution.

Supplementary 4 Figure S4. (a, b) The Fourier Transform Infrared Spectroscopy (FTIR) analysis of the (a) niacin Cu-MOFs and M-Cu-MOFs, (b) niacin Zn-MOFs and M-Zn-MOFs. (c, d) The X-ray diffraction (XRD) patterns of (c) the niacin Cu-MOFs, M-Cu-MOFs, and ALGM, (d) niacin Zn-MOFs, M-Zn-MOFs, and ALGM.

Supplementary 5 Figure S5. The release condition for the (a) niacin Cu-MOFs and M-Cu-MOFs, (b) niacin Zn-MOFs and M-Zn-MOFs in PBS solution. Niacin was used to stimulate the release of metal ions.

Supplementary 6 Figure S6. The biocompatibility of the microcapsules with different (a) niacin Cu-MOFs encapsulated concentrations and (b) niacin Zn-MOFs encapsulated concentrations.

Supplementary 7 Figure S7. The degradation rate of alginate microcapsules in PBS with different* E. coli* concentrations.
