# Supplementary figures and images for: Microfluidic Electrospray Niacin Metal-Organic Frameworks Encapsulated Microcapsules for Wound Healing
Source: Research (Wash D C). 2019 Apr 22;2019:6175398. doi: 10.34133/2019/6175398 (PMC6750103; doi:10.34133/2019/6175398)

## Slide 1
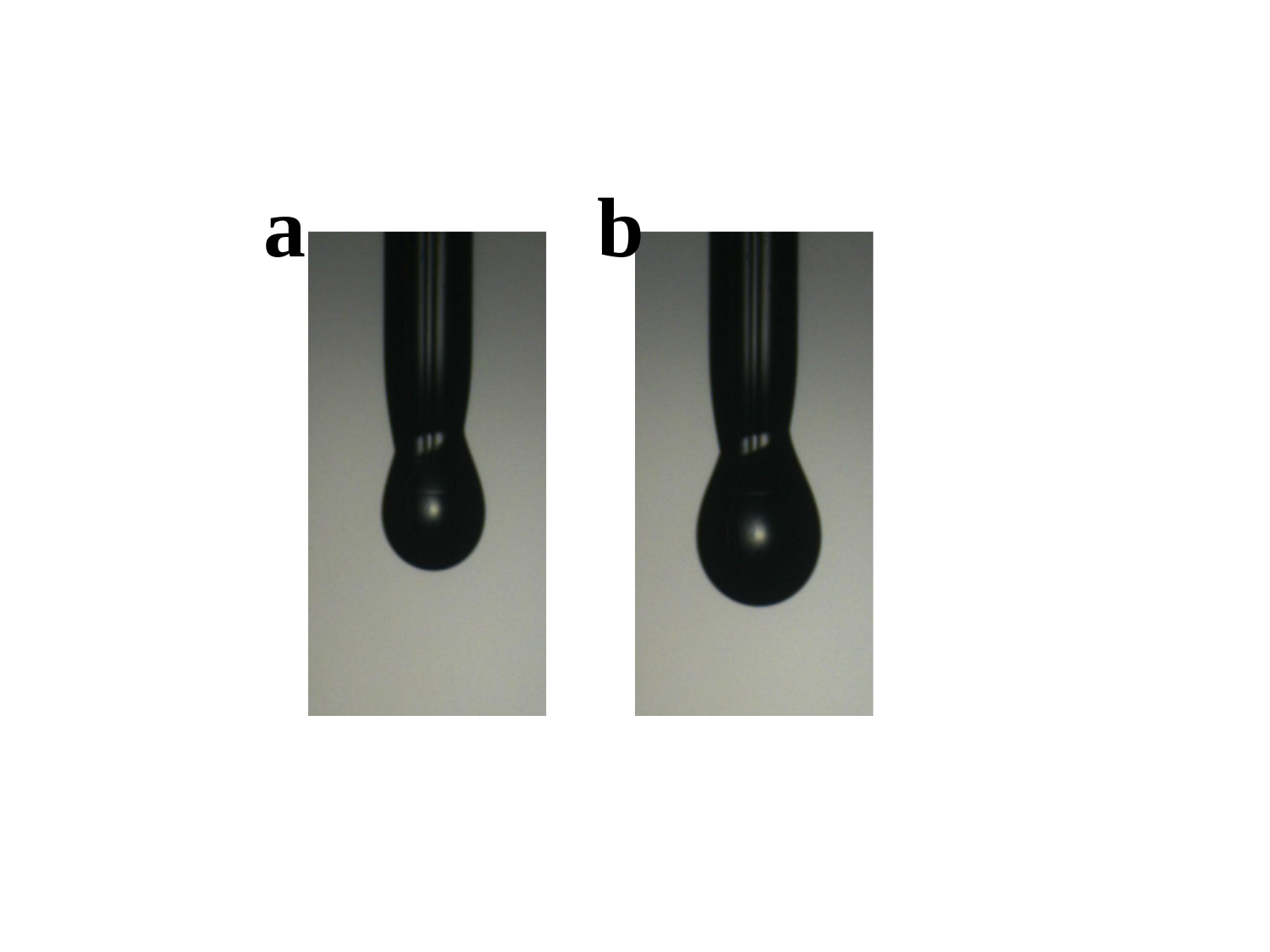

a
b

Supplement: Supplementary 1 — Figure S1. The real-time images of the microfluidic electrospray process of the niacin MOFs encapsulated microcapsules. [file 6175398.f1.pptx]

## Slide 1
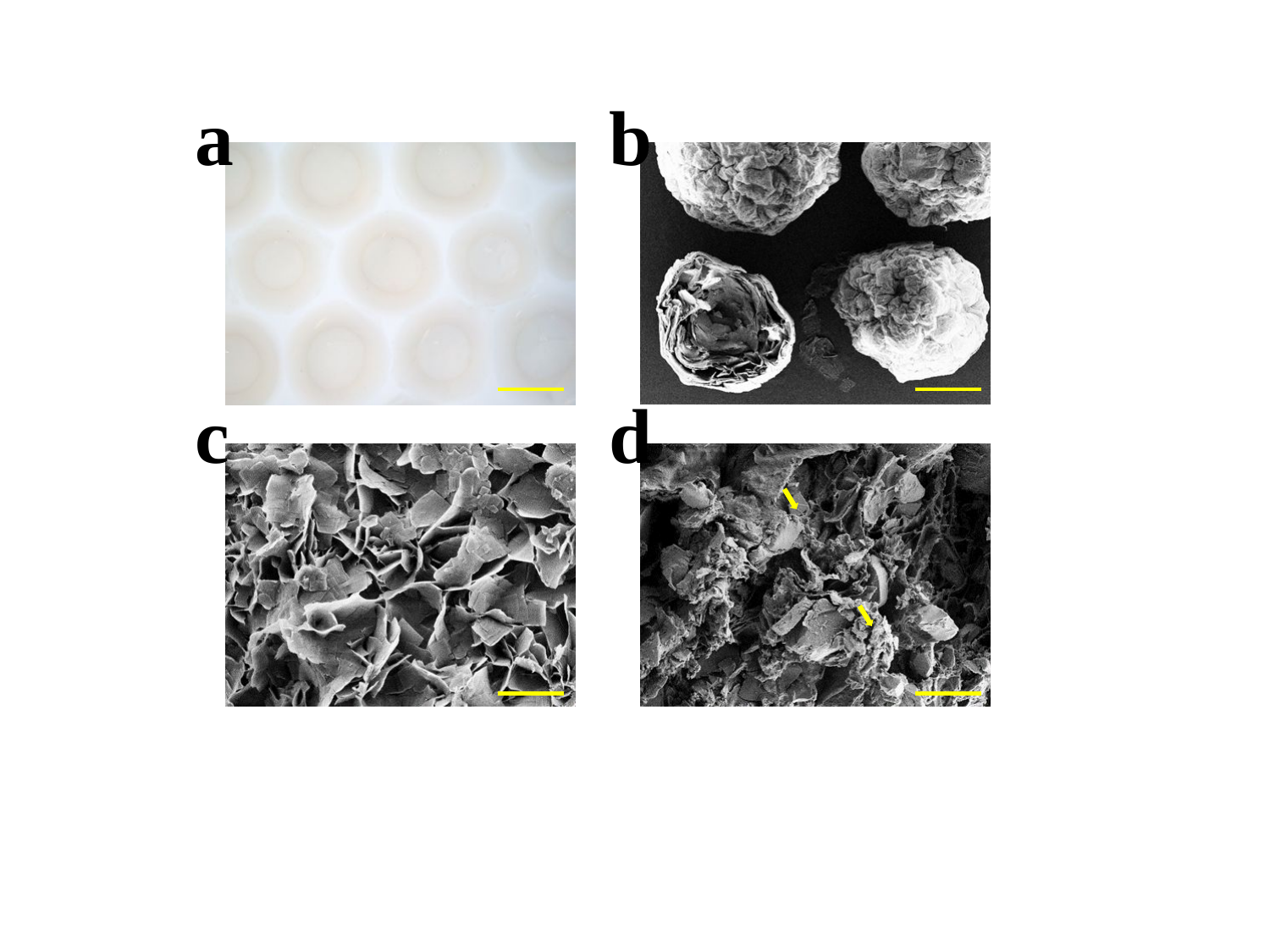

a
b
c
d

Supplement: Supplementary 2 — Figure S2. The preparation of the niacin Zn-MOFs encapsulated microcapsules. (a) Microscope image of the microcapsules. (b-d) Scanning electron microscope (SEM) images of the (b) niacin Zn-MOFs encapsulated microcapsules, (c) niacin Zn-MOFs, and (d) niacin Zn-MOFs inside the microcapsules (indicated with yellow arrows). Scale bar in (a) is 300μm, in (b) is 100 μm, in (c) is 2 μm, and in (d) is 10μm. [file 6175398.f2.pptx]

## Slide 1
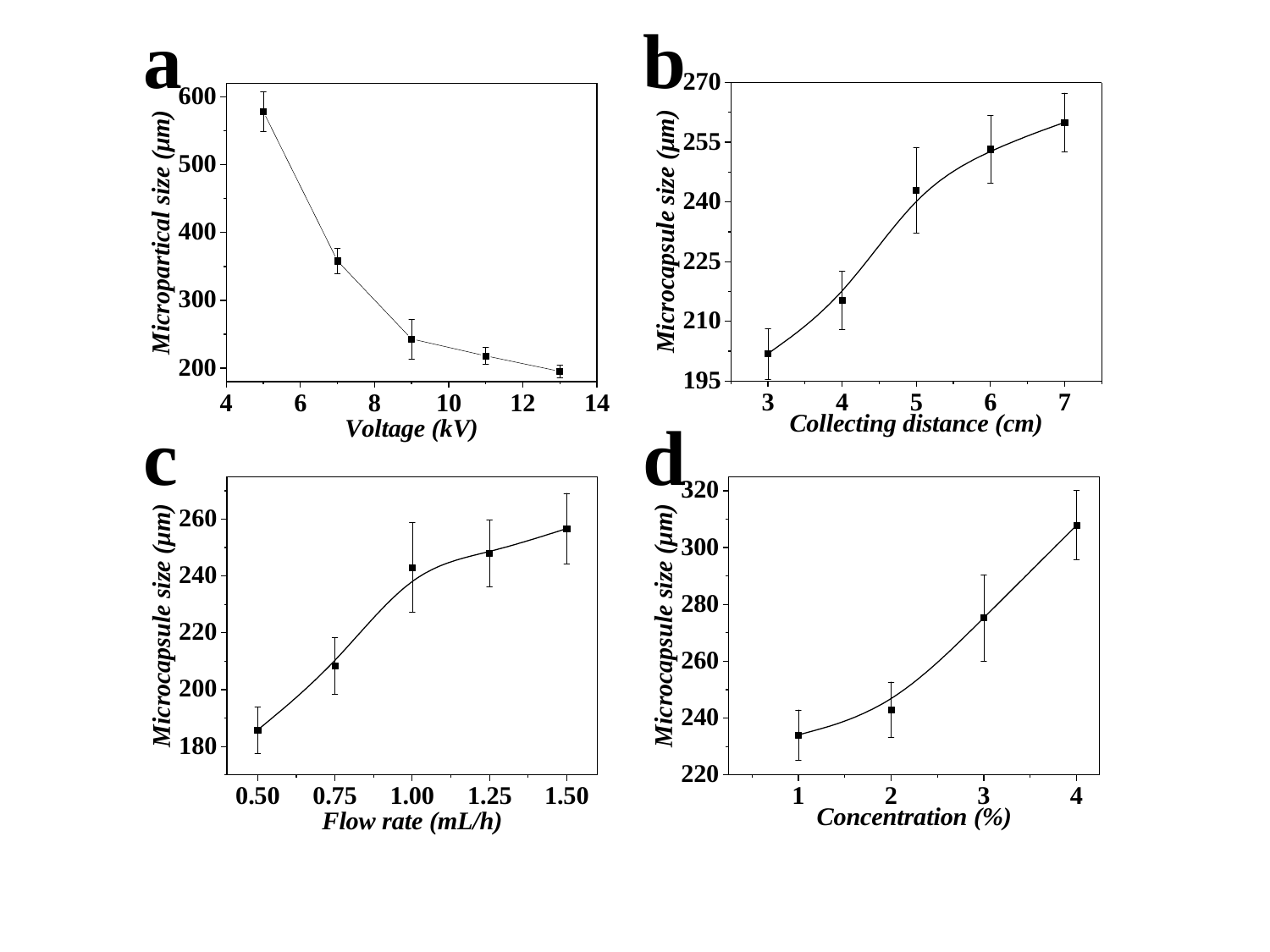

b
a
c
d

Supplement: Supplementary 3 — Figure S3. The relationship between the microcapsule size and (a) voltage, (b) collecting distance, (c) flow rate, and (d) concentration of the alginate solution. [file 6175398.f3.pptx]

## Slide 1
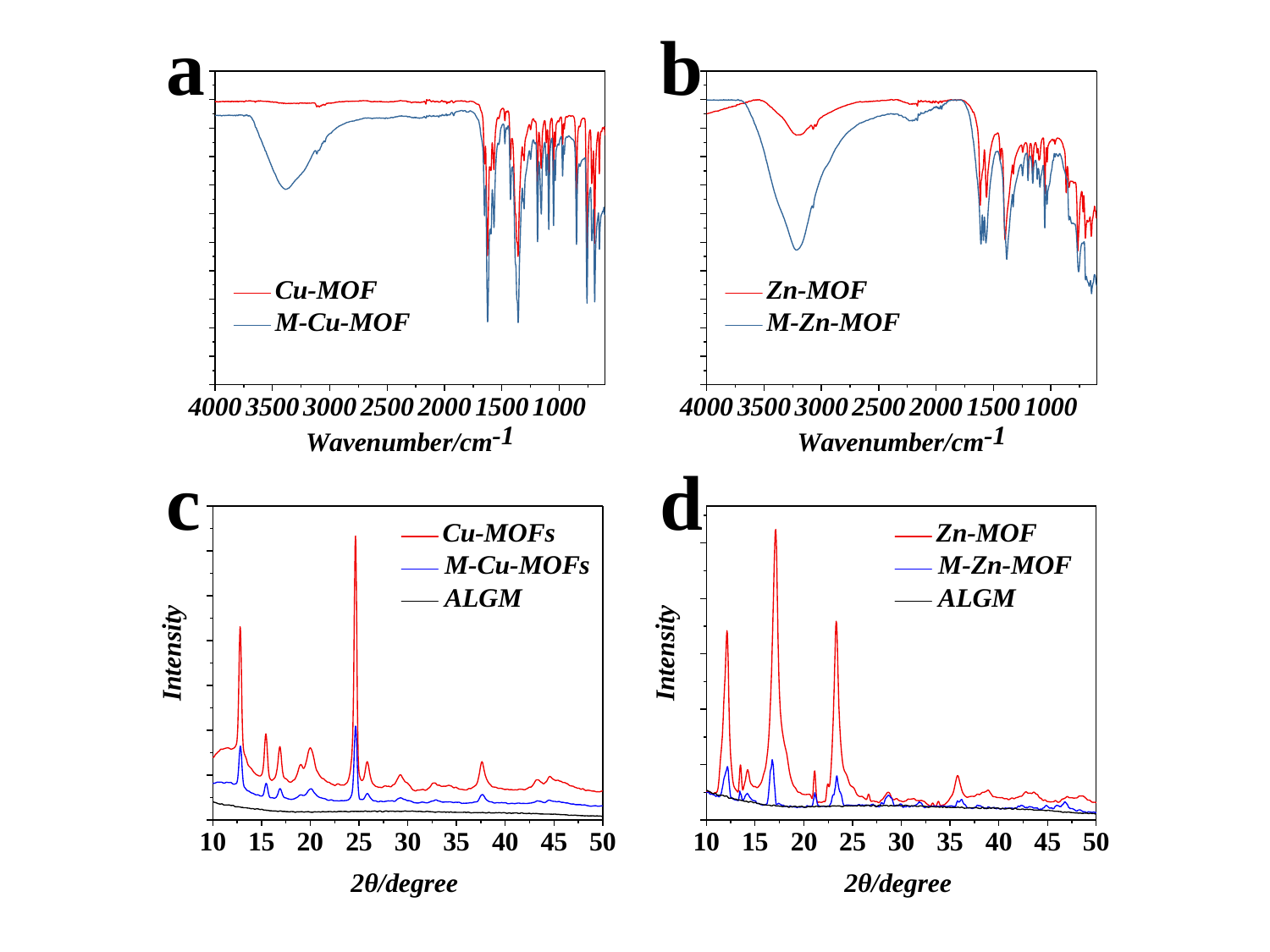

b
a
c
d

Supplement: Supplementary 4 — Figure S4. (a, b) The Fourier Transform Infrared Spectroscopy (FTIR) analysis of the (a) niacin Cu-MOFs and M-Cu-MOFs, (b) niacin Zn-MOFs and M-Zn-MOFs. (c, d) The X-ray diffraction (XRD) patterns of (c) the niacin Cu-MOFs, M-Cu-MOFs, and ALGM, (d) niacin Zn-MOFs, M-Zn-MOFs, and ALGM. [file 6175398.f4.pptx]

## Slide 1
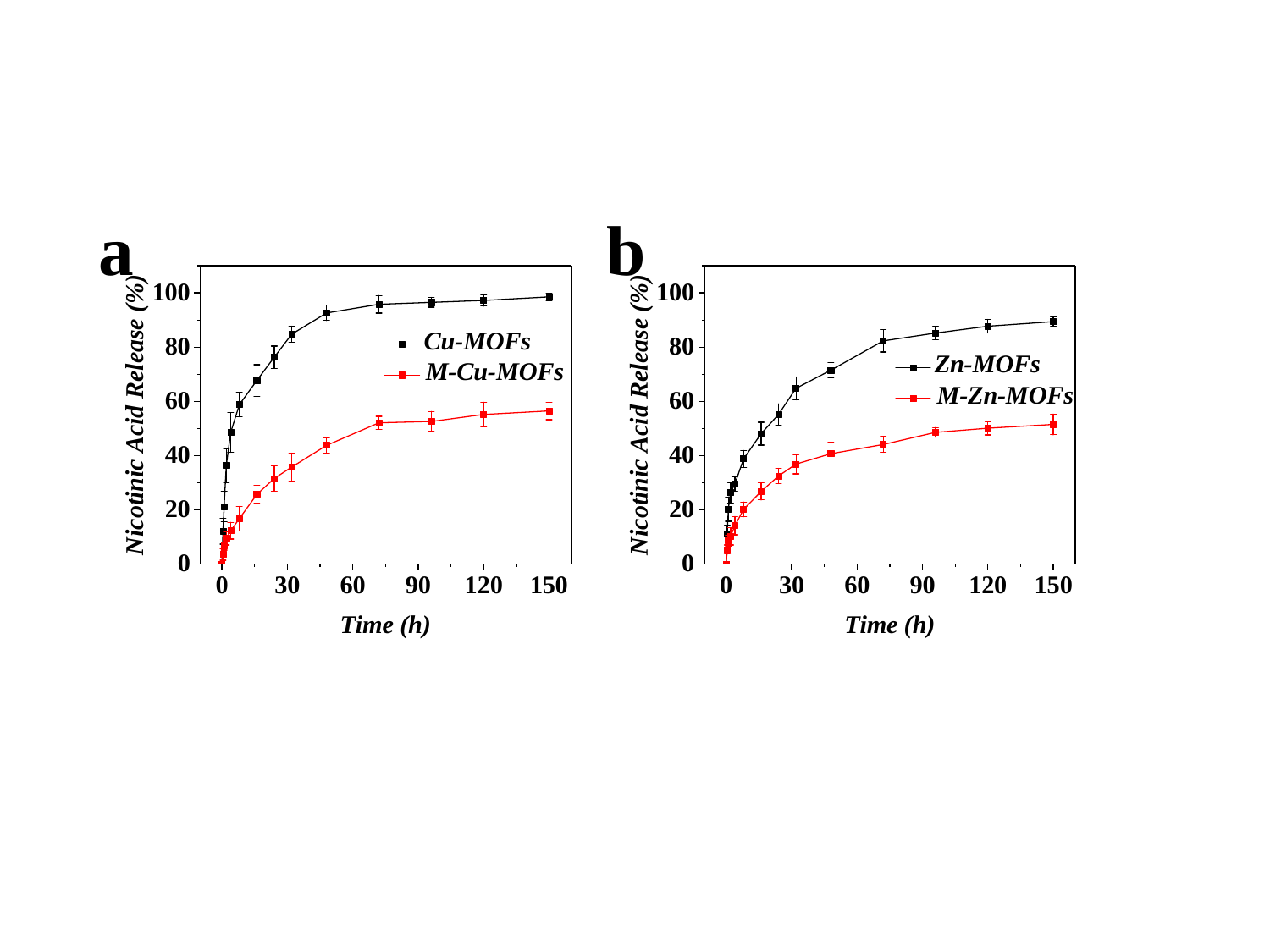

a
b

Supplement: Supplementary 5 — Figure S5. The release condition for the (a) niacin Cu-MOFs and M-Cu-MOFs, (b) niacin Zn-MOFs and M-Zn-MOFs in PBS solution. Niacin was used to stimulate the release of metal ions. [file 6175398.f5.pptx]

## Slide 1
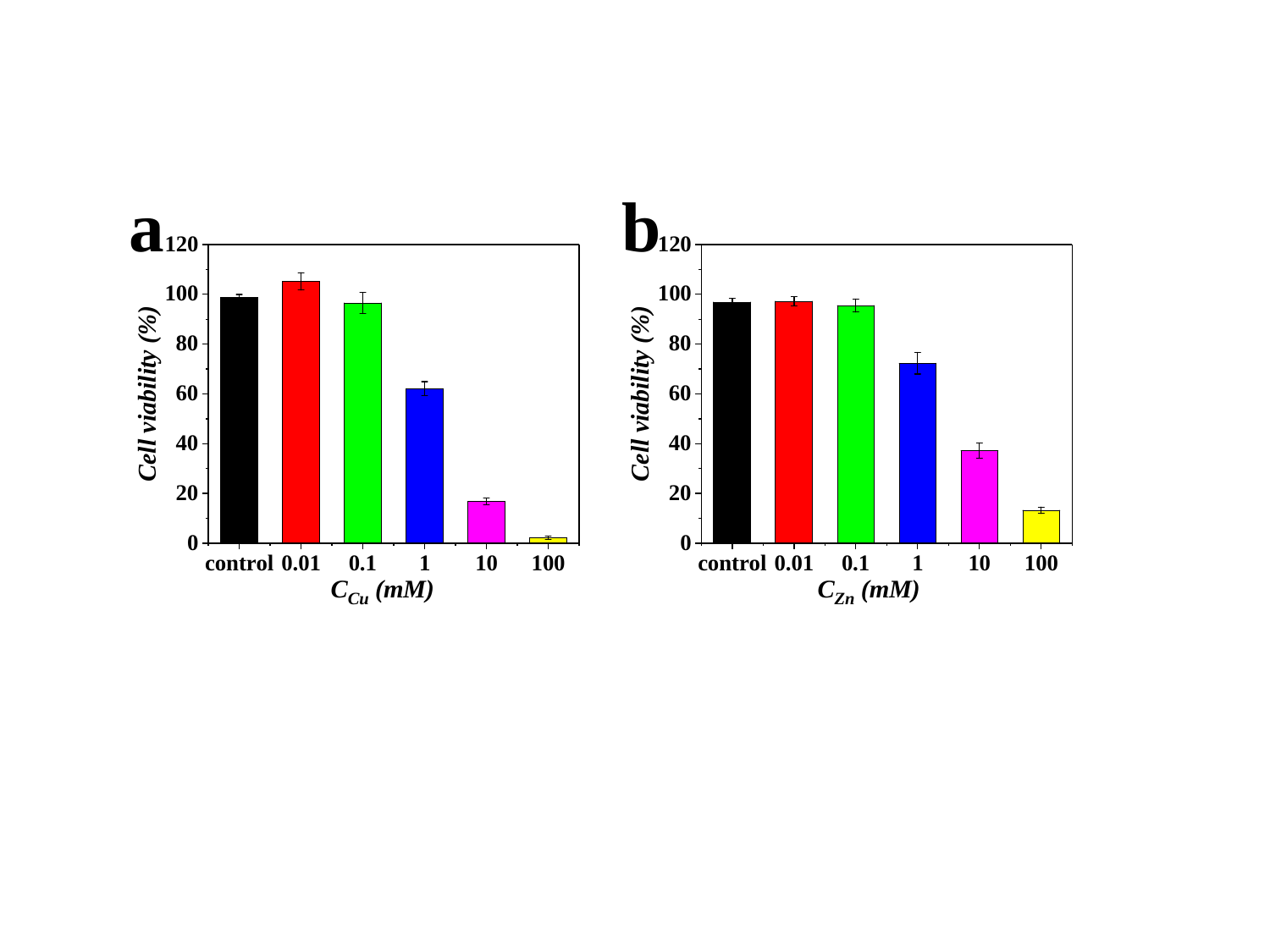

a
b

Supplement: Supplementary 6 — Figure S6. The biocompatibility of the microcapsules with different (a) niacin Cu-MOFs encapsulated concentrations and (b) niacin Zn-MOFs encapsulated concentrations. [file 6175398.f6.pptx]

## Slide 1
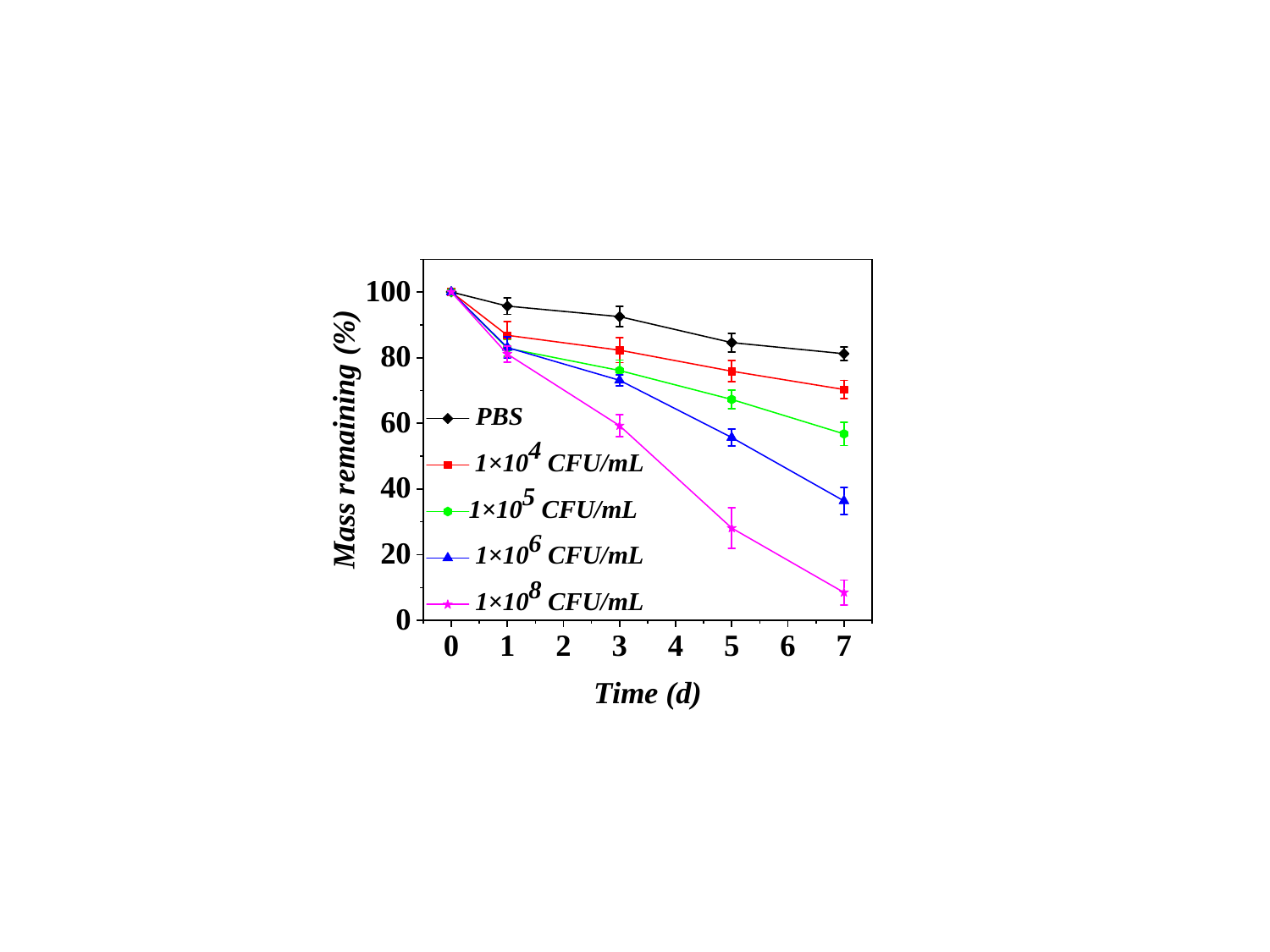

Supplement: Supplementary 7 — Figure S7. The degradation rate of alginate microcapsules in PBS with different E. coli concentrations. [file 6175398.f7.pptx]
